# Mortality Among Patients Discharged From an Acute Psychiatric Department: A 5-Year Prospective Study

**DOI:** 10.3389/fpsyt.2020.00816

**Published:** 2020-08-18

**Authors:** Astrid Prestmo, Karina Høyen, Arne Einar Vaaler, Terje Torgersen, Ole Kristian Drange

**Affiliations:** ^1^ Department of Østmarka, Division of Mental Health Care, St. Olavs Hospital, Trondheim University Hospital, Trondheim, Norway; ^2^ Department of Mental Health, Norwegian University of Science and Technology, Trondheim, Norway; ^3^ Orkdal District Psychiatric Centre, St. Olavs Hospital, Trondheim University Hospital, Trondheim, Norway

**Keywords:** psychiatric inpatients, excess mortality, post discharge suicide, standardized mortality ratio, acute psychiatric department

## Abstract

The primary aim of the study was to explore the post discharge standardized mortality ratio of patients from an acute psychiatric department in Norway. The secondary aims were to explore if the standardized mortality ratio is still increasing and to examine the causes of death in the defined population. We conducted a 5-year prospective study among patients admitted to an acute psychiatric department with catchment area responsibilities. A total of 380 patients were included in the study, and the number and causes of deaths were obtained from the Norwegian Cause of Death Registry. Excess mortality was found for the patient group. The standardized mortality ratio for all causes of death was 6.7 (95% CI, 4.6–8.8). The study finds an increased standardized mortality ratio relative to a previous corresponding study in Norway, and the suicide risk was especially elevated the first 2 years after discharge.

## Introduction

Studies from all over the world have found excess mortality for psychiatric patients (1–5). The standardized mortality ratio (SMR) in psychiatric populations has varied from being 1-fold to 20-fold higher than in the general population, depending on psychiatric diagnosis, patient population, and causes of death (6–8). Most psychiatric diagnoses carry an elevated mortality risk (4, 9, 10), and the highest risk of premature death has been found among patients with substance use disorders (3, 5, 7, 11, 12) or organic mental disorders (13, 14).

Several studies from the Nordic countries have also shown excess mortality rates in patients with psychiatric disorders, especially among patients discharged from psychiatric hospitals (2, 15, 16). A Norwegian study conducted by Nome and Holsten (16) showed an overall SMR of 2.9 [95% confidence interval (CI), 2.5–3.1] and 2.2 (95% CI, 1.9–2.4) for male and female patients, respectively, discharged from a psychiatric hospital from 1985 to 2003. Further, the study showed an alarming increase in SMR for both genders during the study period. The increasing SMR was explained by both a decreased mortality in the general population, and an increased mortality in the study population.

The excess mortality after discharge from psychiatric facilities has been explained by both natural and unnatural causes (17, 18). The most common natural causes of death in psychiatric patients are cardiovascular and respiratory diseases (13, 18, 19). Some studies have suggested that patients with mental disorders receive a lower quality of healthcare in the treatment of their somatic disorders (10, 20). The excess mortality by natural causes may also be explained by several other reasons including that the population is vulnerable with limited ability to utilize information about health promotion (21, 22), under-diagnosis and under-treatment of somatic conditions among mentally ill patients (23, 24), adverse effects of psychotropic drugs (25, 26), and common genetic risk factors for somatic and psychiatric disorders (13).

Among unnatural causes of death, several studies have reported a considerable increased risk of suicide after discharge from psychiatric hospitals (2, 27–30). Results from a review from the United States found a pooled estimated discharge suicide rate of 484 per 100,000 person years. The suicide rate was roughly 100 times the global suicide rate during the first 3 months after discharge, studies with a follow-up period of 3–12 months showed suicide rates that were 60 times the global rate, and studies with a follow-up period of 5–10 years had rates 30 times that of the global rate (30). These figures represent a significantly higher and more enduring risk that was generally believed, as stated by the authors, the figures after 3 months are more than three times the suicide rates presented in comparable studies (30, 31). Studies from Nordic countries with similar follow-up durations have also reported high suicide rates after discharge from psychiatric hospitals (29, 32–34). Qin and Nordentoft found that suicide risk was especially elevated both the first week after admission and the first week after discharge (35).

Other causes of unnatural death in psychiatric patients are the heightened risk of accidents and drug overdoses (36). Several studies have reported higher mortality in males than in females (7, 16, 37), but some studies have found that the relative risk of death for women is higher than for men because of the lower mortality rate for women in the general population (15, 38).

In Norway and most western countries, the mental health systems have undergone major organizational and ideological changes in the last decades (39). The number of inpatient beds has been reduced, while the access to outpatient treatment has substantially increased. Some authors have suggested that this may have contributed to an increase in premature death among psychiatric patients, but the literature is not consistent (1, 13, 40).

In the present prospective cohort study, we followed the mortality in a sample of adult patients discharged from an acute psychiatric department in Norway during a 5-year study period using the Norwegian Cause of Death Registry.

The primary aim of the study was to explore the post-discharge SMR in acute psychiatric inpatients. The secondary aims were to find if the SMR is still increasing in Norway compared with Nome’s study of a similar population in the period from 1985 to 2003 and to examine the causes of death, including the frequency of suicides in this population, to identify where to take preventive measures.

## Methods

### Setting

In this prospective study, patients ≥18 years were included at admittance to the acute psychiatric department at St. Olavs hospital, Trondheim, Norway. Norwegian acute psychiatric services are catchment area-based, publicly funded, and available to everyone. The catchment area consists of both urban and rural parts and is covering 310,000 residents. The catchment area is typical for Norway when it comes to number of psychiatric beds,^1^ level of drug/alcohol abuse, and annual number of suicides.^2^ The patients were included from September 2011 through March 2012 as a part of a study assessing agitation in acute psychiatric departments.^3^ The admission rates during 2011 and 2012 were not different between the winter (average monthly admission rate from December 2011 to February 2012 was 179.7) and summer (average monthly admission rate from June to August 2012 was 180.7) seasons.

### Participants

Seven hundred and sixty patients were admitted in the study period, all patients admitted were asked to participate in the study, and 380 (50%) gave informed consent to participate. One hundred and thirty-one (34.5%) out of the 380 included patients had their first admission during the study period. Suicidality was part of the reason for admission among 72% of the participants. Three hundred and forty-two patients were admitted once, and 38 patients had several admissions during the study period (**Table 1**).

**Table 1:** Characteristics of included participants admitted once (n = 342) and several times (n = 38) in the study period.

	Number of admissions = 1		Number of admissions < 1	
	n (%)	M (SD)	n (%)	M (SD)
**Sex** (Male)	180 (52.3)		16 (44.7)	
**Age**		38.5 (14.9)		42.4 (15.6)
**Diagnosis**				
Affective disorders	121 (35.8)		11 (28.9)	
Substance use disorders	70 (20.6)		10 (26.3)	
Schizophrenia spectrum disorders	46 (14.0)		5 (7.9)	
Neurotic, stress-related disorders	30 (8.7)		4 (10.5)	
Personality disorders	32 (8.4)		3 (10.5)	
Organic disorders	15 (4.7)		3 (5.3)	
Other disorders	28 (8.2)		2 (10.5)	

Most patients who were not included were admitted at night and discharged the following morning before inclusion could be performed. In the case of several admissions for the same patient, we used data from the index admission only. The consent from the Regional Ethical committee limited the inclusion to maximum 400 unique patients.

### Data Sources

Diagnoses according to the International Classification of Diseases 10^th^ revision (ICD-10) Criteria for Research (41) were set in a consensus meeting, in which at least two specialists in psychiatry participated, and at least one of whom had personally examined the patient.

Information on number and causes of deaths were obtained from the Norwegian Cause of Death Registry at the National Institute of Public Health.^4^ Linking the patients to the National Cause of Death Registry was done using their personal identification number.

Data on mortality was based on all recorded deaths within 5 years after index admission. The follow-up time was calculated from the date of discharge until death within 5 years after discharge for every unique patient. The deaths were categorized as due to natural or unnatural causes. Natural causes of death were defined as those with ICD-10 codes A00-R99. Unnatural causes of deaths were defined as those with ICD-10 codes V01-Y89 (accidental, intentional, and undetermined causes of death).

The medical records of the patients who had died during the follow up period were finally reviewed by two psychiatrists, independent of each other, to increase the quality of the categorization.

The age-, gender-, and cause-specific mortality rates for each year in the catchment area were provided from Statistics Norway (2018)^5^ and used to calculate the number of expected deaths.

### Ethics

Information about the study was given by an experienced specialist in clinical psychology or psychiatry to ensure that the patients had the capacity to give informed consent.

All included patients gave written informed consent to participate in the study, including linkage of data to health registers. The study was approved by the Regional Committee for Medical and Health Research Ethics, Central Norway (ref. 2011/137) and conducted according to the Declaration of Helsinki. The study is registered in the ClinicalTrials.gov (ref. NCT01415323).

### Statistical Analyses

Statistical analyses were performed with the data analysis tool set in Microsoft EXCEL 2015 and the statistical packages SPSS 24 and R 3.4.0 for Windows.

We used descriptive statistics to present mean age in the study population, median age at death, and interval from first admission to death.

Counts and proportion of deaths were calculated within the total study population and within different age (under or above 70 years), gender (men and women), and diagnosis (substance use disorder, schizophrenia spectrum disorder, and affective disorder) groups. We used a Chi-square test to compare the proportion of deaths due to all causes, natural causes, and unnatural causes between the mentioned groups. The age-specific mortality rates were modeled on the Poisson distribution.

To compare mortality for the study population with the general population in the catchment area, the SMR with 95% CI was calculated. The estimates of population mortality were found by using the official mortality rates for cohorts of individuals born in a specific year who died in a given year. Patient mortality was then compared with the corresponding mortality estimates in the general population. SMR was calculated by dividing the number of observed deaths in the study population by the number of expected deaths in the general population.

### Comparison With the Nome Study

Nome et al. (16) had a 20-year follow up period (1985–2003), subdivided into 5-year intervals. The mortality rates were calculated for every 5-year period with one exception (i.e., the last period was slightly over 3 years). We compared the SMR in our study with the SMR in the four intervals in Nomes’ study. Nome only included patients who had their first admission in the study period. We asked all admitted patients to participate in the study, but also explored patients with their first admission.

## Results

The cohort consisted of 380 patients with a mean age of 38.9 years (SD, 15; range, 18–83), and 51.6% were men. The median duration of hospitalization was 9 days (range, 1–91 days). The most prevalent primary diagnoses were affective disorders (34.7%), substance use disorders (21.1%), and schizophrenia spectrum disorders (13.4%) (**Table 2**). The distribution of sex and age in the non-participating population (N = 380, mean age of 40.7 (SD, 17.3), and 52.8 were men) were comparable with the study population. The most prevalent diagnoses in the non-participating population were schizophrenia spectrum disorders (24.8%), substance use disorders (19.1%), and affective disorders (18.3%).

**Table 2:** Characteristics of patients admitted to an acute psychiatric department from 2011 to 2012.

	Male	Female	Total
	[n (%)]	[n (%)]	[n (%)]
**Demographic variables**Age			
<20	6 (3.1)	7 (3.8)	13 (3.4)
20–29	50 (25.5)	69 (37.5)	119 (31.3)
30–39	47 (24)	33 (17.9)	79 (20.8)
40–49	46 (23.5)	34 (18.5)	84 (22.1)
50–59	23 (11.7)	26 (14.1)	49 (12.9)
60–69	15 (7.7)	10 (5.4)	26 (6.8)
70–79	6 (3.1)	5 (2.7)	12 (2.1)
80–89	3 (1.5)	0	3 (0.8)
Total	196	184	380
%	51.6	48.4	100
Mean age (SD)	40.6 (15.0)	37.3 (14.9)	38.9 (15)
**Clinical variables** Diagnosis			
Affective disorders (F30-39)	59 (30.1)	73 (39.7)	132 (34.7)
Substance abuse disorders (F10-19)	60 (30.6)	20 (10.9)	80 (21.1)
Schizophrenia spectrum disorders (F20-29)	30 (15.3)	21 (11.4)	51 (13.4)
Neurotic, stress-related disorders (F40-48)	17 (8.7)	17 (9.2)	34 (8.9)
Personality disorders (F60-69)	6 (3.1)	28 (15.3)	34 (8.9)
Organic disorders (F00-09)	8 (4.1)	10 (5.4)	18 (4.7)
Other disorders	16 (8.2)	15 (8.2)	31 (8.2)
Admission status			
Voluntary admission	150 (76.5)	142 (77.2)	292 (76.8)
Compulsory admission	46 (23.5)	42 (22.8)	88 (23.2)
Duration of stay in days [mean (SD)]	8.3 (8.9)	9.6 (10.3)	9.0 (9.6)

One hundred and thirty-one participants had the index admission as their first psychiatric admission. In this group, mean age was 36.8 (SD, 15.8; range, 18–83), and 55.7% were men, and the median duration was 8 days (range, 1–38). The most prevalent primary diagnoses were affective disorders (42.7%), substance use disorders (20.6%) and neurotic, stress-related, and somatoform disorders (13%).

### Mortality in the Sample

Thirty-nine patients (10.3%) died within the 5-year follow-up period. The mean age at death was 49.6 (SD, 18.4; range, 20–83) years. Significantly more male patients died than female patients (**Table 3**).

**Table 3:** Characteristics of patients who died during the 5 years after discharge from an acute psychiatric department.

	Male	Female	All
	(n (%))	(n (%))	(n (%))
**Age**			
20–29	4 (13.8)	3 (30)	7 (17.9)
30–39	5 (17.2)	2 (20)	7 (17.9)
40–49	6 (20.7)	0	6 (15.4)
50–59	2 (6.9)	3 (30)	5 (12.8)
60–69	8 (27.6)	1 (10)	9 (23.1)
70–79	1 (3.4)	1 (10)	2 (5.1)
80–89	3 (10.3)	0	3 (7.7)
Total	29	10	39*
%	74.4	25.6	100
Mean age (SD)	51.2 (18.1)	45.8 (19.4)	49.6 (18.4)
**Diagnosis**			
Affective disorders (F30-39)	6 (20.7)	4 (40)	10 (25.6)
Substance use disorders (F10-19)	14 (48.3)	1 (10)	15 (38.5)
Schizophrenia spectrum disorders (F20-29)	2 (6.9)	2 (20)	4 (10.3)
Neurotic, stress-related disorders (F40-48)	2 (6.9)	1 (10)	3 (7.7)
Personality disorders (F60-69)	1 (3.4)	1 (10)	2 (5.1)
Organic disorders (F00-09)	4 (13.8)	1 (10)	5 (12.8)
**Admission status**			
Voluntary admission	24 (82.8)	8 (80)	32 (82.1)
Compulsory admission	5 (17.2)	2 (20)	7 (17.9)
**Duration of stay in days (SD)**	8.8 (10.5)	10.0 (7.8)	9.1 (9.8)

*(p = 0.03, Pearson’s Chi squared test).

For patients younger than 70 at index admission, the mortality rate was 9.3% in total. The mortality rate was significantly higher for men (13.4%) than for women (5%) (p < 0.0001).

### Excess Mortality 5 Years After Discharge

The SMR for all causes of death is shown in **Table 4**.

**Table 4:** Standardized mortality ratios (SMR) and confidence intervals (95% CI) of all patients and stratified by sex and diagnosis.

	SMR	95% CI
**Sex**		
All subjects	6.68	4.58–8.77
Male	6.91	4.40–9.43
Female	6.10	2.31–9.85
**Diagnosis**		
Affective disorders (F30-39)	3.92	1.49–6.35
Substance use disorders (10-19)	12.10	5.97–18.22
Schizophrenia spectrum disorders (F20-29)	10.53	0.21–20.84
Neurotic, stress-related disorders (F40-48)	10.00	0–21.32
Personality disorders (F60-69)	10.81	0–19.16
Organic disorders (F00-09)	5.88	0.7–11.04

SMR for all causes of death was 6.7 (95% CI, 4.6–8.8). When stratified by gender the SMR were 6.9 (95% CI, 4.4–9.4) for male and 6.1 (95% CI, 2.3–9.9) for female. For patients having a substance abuse disorder, the SMR for all causes of death was 12.1 (95% CI, 6.0–18.2), and when stratified by gender the SMR was 11.5 (95% CI, 5.5–17.6) for male and 20 (95% CI, 19.2–59.2) for female (**Table 4**). For patients without a substance abuse disorder, the corresponding results were 4.7 (95% CI, 2.2–7.1) and 6.3 (95% CI, 2.4–10.1) for male and female respectively.

The SMR for patients younger than 70 years was 12.5 (95% CI, 7.5–17.5) for male and 9.3 (95% CI, 3.5–15.1) for female.

SMR for all natural causes of death was 3.6 (95% CI, 2.0–5.3), 3.5 for male (95% CI, 1.6–5.4) and 4.0 for female (95% CI, 0.8–7.3). **Table 4** shows that the highest SMR for death due to natural causes was among patients with substance use disorders.

SMR for all unnatural causes of death was 25.4 (95% CI, 13.3–37.4). When stratified by gender, the SMRs were 25.0 (95% CI, 11.4–38.6) for male and 26.7 (95% CI, 0.5–52.8) for female. Also in the group of patients who died by unnatural causes, the highest SMR found was among patients with substance abuse disorders (**Table 5**).

**Table 5:** Standardized mortality ratios (SMR) and confidence intervals (95% CI) of different causes of death in all patients and patients suffering from affective disorders or substance abuse.

	Patient groups					
	All patients		Affective disorders		Substance abuse disorder	
	SMR	95% CI	SMR	95% CI	SMR	95% CI
**All deaths**						
All subjects	6.68	4.58–8.77	3.92	1.49–6.35	12.10	5.97–18.22
Male	6.91	4.40–9.43	3.64	0.73–6.55	11.52	5.49–17.56
Female	6.10	2.31–9.85	4.44	0.09–8.80	20.00	0–59.20
**Natural deaths**						
All subjects	3.64	2.00–5.28	1.74	0.03–3.44	5.56	1.11–10.00
Male	3.49	1.60–5.38	1.33	0–3.18	5.71	1.14–10.29
Female	4.03	0.80–7.25	2.50	0–5.96	0*	0
**Unnatural deaths**						
All subjects	25.37	13.31–37.44	23.81	2.94–44.68	58.25	20.19–96.31
Male	25.00	11.41–38.59	20.00	0–42.63	57.14	17.54–96.74
Female	26.67	0.53–52.80	33.33	0–79.53	66.67	0–197.33
**Suicides**						
All subjects	55.32	25.25–85.39	53.33	1.07–105.60	66.67	1.33–132.00
Male	54.55	18.91–90.18	40.00	0–95.44	80.00	1.60–158.40
Female	57.14	1.14–113.14	80.00	0–190.87	0*	0

*No patients died in this group.

For patients who died by suicide, the SMR was 55 (95% CI, 18.9–90.2) for male and 57 (95% CI, 1.1–113.1) for female. The highest risk of suicide was among men having a substance use disorder (**Table 5**).

### Excess Mortality 2 Years After Discharge

We found that the SMR for all causes of death during a 2-year follow-up was 8.3 (95% CI, 4–12.7) and 6.1 (95% CI, 0.1–12) for males and females, respectively. The risk of dying by suicide 2 years after discharge was roughly 100 times higher than in the general population and was the highest for men [SMR, 95.7 (95% CI, 33.2–158.3); m/f: 106 (95% CI, 27.5–184.6)/71.4 (0–170.4)]

### Excess Mortality of Patients With Their First Admittance

One hundred and thirty-one participants were admitted for the first time. We found that the SMR for all causes of death in this group was 5.2 (95% CI, 3.1–7.3). When stratified by gender, the SMR was 4.1 (95% CI, 1.4–6.8) for males and 10.5 (95% CI, 0.2–20.8) for females. Nome et al. (16) only included patients upon their first admittance. In the present study, the SMR for patients who were being admitted for the first time were lower than for the total study group but still exceeded the estimates from the study of Nome et al. (16), except for the last period.

### Mortality Compared With Nomes Study

The estimates of the SMR all causes of death in the present study exceed all estimates from the Nome and Holsten (16) study. The last period in the study of Nome had a follow-up period slightly over 3 years after. We therefore compared the SMR 3 years after discharge and found that our numbers exceeded the SMR from Nome’s study. However, the CI for males and all patients slightly overlap. We also found that the SMR for patients younger than 70 years at discharge had a higher SMR than the whole study group.

### Causes of Death in the Sample

The causes of death were categorized by natural (ICD-10 codes A00-R99) and unnatural causes (ICD-10 codes V01-Y89). Nineteen patients died by natural causes, 17 patients died by unnatural causes, and three patients had an unknown cause of death (**Table 6**). The most prevalent causes of natural death were pulmonary diseases, cancer, and cardiovascular diseases. Among unnatural causes of death, the most prevalent diagnosis was intentional self-poisoning, intentional self-harming by strangulation, and intentional drowning or submersion (**Table 5**). Thirteen of the patients who died by unnatural causes were classified as having died due to suicide (X6n).

**Table 6:** Causes of death in the cohort (n = 39).

	n (%)	M (SD)
**Natural causes**	**19** (48.7)	
Age		62.3 (14.8)
Sex (male)	12 (68.4)	
Pulmonary diseases	6 (31.6)	
Cancer diseases	4 (21)	
Cardiovascular disease	4 (21)	
Bacterial meningitis	1 (5.3)	
Anorexia	1 (5.3)	
Epilepsy	1 (5.3)	
Multiple sclerosis	1 (5.3)	
Cirrhosis	1 (5.3)	
**Unnatural causes**	**17** (43.6)	
Age		36.0 (11.5)
Age (suicide)		35.0 (11.6)
Sex (male)	13 (76.4)	
Intentional self-poisoning	7 (53,8)	
Intentional self-harming by strangulation	2 (15.4)	
Intentional self-harming by drowning and submersion	2 (15.4)	
Intentional jumping or lying before moving object	1 (7.8)	
Intentional self-harming by jumping from a high place	1 (7.8)	
Accidental poisoning	3 (23.1)	
Exposure to unspecified factor	1 (7.8)	
**Uknown causes of death**	**3** (7.7)	

The median age of natural and unnatural death was 62.3 (SD, 14.8) and 36 (SD, 11.5), respectively. The median age at death for patients with suicide was 38.8 (SD, 12.1)/28.0 (SD, 6.5) for men/female.

## Discussion

The present study indicates a mortality gap between patients discharged from acute psychiatric departments and the general population in Norway. We found a standardized mortality rate (SMR) of seven in the total sample during 5 years of follow-up. The SMR was 11 among those below 70 years of age and five among those who had their first admittance. Among the diagnostic groups, the highest SMRs were found for patients with substance use disorders and schizophrenia spectrum disorders. In our sample of 380 patients, 19 died by natural causes and 17 died by unnatural causes during follow-up. The most prevalent natural causes were pulmonary diseases, cancer, and cardiovascular disease, while the most prevent unnatural cause was suicide.

Our finding of an increased post-discharge SMR among patients admitted to psychiatric departments corresponds to findings in several previous studies (15, 39, 42–44). However, a SMR of seven is considerably higher than in a previous corresponding study from Norway (16).

Nome and Holsten (16) described the post-discharge SMR from psychiatric departments among patients having their first admissions. Follow-up times were 5 years for three periods (1985–1989, 1990–1994, and 1995–1999) and 3 years for one period (2000-03). We compared the SMR in our study with the SMR in the four intervals in Nomes’ study. In the present study, we included patients for 7 months, and follow-up time was 5 years. The similarities between the studies are that they are done in the same country with the same health care system. There are, however, a number of differences between the studies. Nomes study has a higher participant rate, longer follow-up time, and used numbers only from patients with their first admission. In the present study, we asked not only all admitted patients to participate in the study but also explored patients with their first admission. The practice of only including patients giving written informed consent differs from the study of Nome et al., which is explained by restrictions in the approval from the research ethics committee.

The SMRs in our study exceed all figures in the study of Nome and Holsten, even when we restrict our analyses to patients being admitted for the first time. Our study cannot determine whether the apparently increasing SMR is due to a *de facto* increased mortality gap, differences in the study populations (i.e., acute psychiatric departments versus all psychiatric departments), or other kinds of bias.

The SMR of 11 among patients below 70 years of age is higher than in similar studies (9, 16). When exploring the patients who had their first admittance, we found lower SMR figures than for the total study group but still excess mortality. This is not in agreement with some similar studies finding higher mortality for patients with their first admittance (8, 45, 46). Reasons for this discrepancy can be easy access to out-patient treatment in Norway, and the fact that many patients had received out-patient treatment before first admission, which may have a positive impact on lowering the suicide risk. Another reason can be that the threshold for admission is lowered because of access to outpatient’s treatment, so the patients can be reached before they develop severe mental symptoms. Our findings of the highest mortality rates among patients diagnosed with substance use disorders and schizophrenia harmonizes with previous results (2, 7, 18).

A number of studies have found increased mortality due to unnatural causes, especially suicide, after discharge from psychiatric facilities (2, 29, 30). The present study confirms that finding, with the risk of dying by suicide 2 years post discharge being roughly 100 times higher than in the general population. The risk decreased after 2 years but still remained high for the next 3–5 years. In addition, the suicide rate was highest in men and might indicate that the mortality rate for men was higher in the first 2 years compared with the next 3–5 years after discharge. However, the CIs slightly overlap. For females, the mortality risk was approximately the same in the whole study period. Past studies have indicated that patients with substance use disorders have shorter length of stay (47–49). There have also been indications of an association between length of stay and risk of post discharge suicide (50). The potential association between length of stay and risk of suicide among participants with substance use disorder has not been addressed in the current study due to limited study size. A Norwegian register study confirms that a high number of suicide victims are in contact with psychiatric health care prior to the act (51). This indicates that, with proper assessments and screening instruments, it should be possible to pinpoint selected patients or groups of patients and increase the effectiveness of treatment and care. The World Health Organization (52) has recommended that suicide prevention for people with mental disorders should have high priority, and many countries have developed suicide preventive strategies. In spite of this, it seems like the suicide figures are increasing (30)

The SMR for all natural causes of death was, as expected, lower than for unnatural causes of death (2, 3, 7), but the mortality rate was still three- to four-fold higher than in the general population. The mortality for all natural causes of death was approximately the same during the follow-up period. Efforts to reduce the burden of mortality among patients with mental disorders need to also address the problem of natural causes (53, 54). People with mental disorders need better coordinated and integrated care between the mental health and medical systems (4).

The strengths of this study are the prospective design, the catchment based, publicly funded health care system, and the registers in Norway that make it possible to find and follow every patient after discharge.

Our study population is small, which provides some methodological limitations in terms of generalizing the findings. Mortality in a group of patients is complex and depends on multiple factors and their mutual influence, such as mental health services availability, health level, mortality in the general population, the age and gender distribution of the patient group, and the period for the first admission to hospital. In addition, differences in stages of an illness, medication, and outpatient contact make it difficult to compare mortality. Our figures are higher than in similar studies, but differences could be influenced by heterogeneity in the mentioned factors between study populations. The low population size and participation rate could also limit the generalizability of our results. Specifically, schizophrenia was more common in the non-participating population and is associated with a high SMR, which could indicate that our study underestimates the SMR at acute psychiatric departments.

In conclusion, the post-discharge SMR within this sample indicates an increase in mortality in psychiatric patients. The post-discharge risk of suicide is high, especially 2 years after discharge. Men with a substance abuse disorder carry the highest suicide mortality risk. Summarized, our findings indicate that in spite of having a publicly funded psychiatric health service and relatively easy access to outpatient treatment, the post-discharge mortality gap seems to be still increasing in Norway. We must focus on high-risk populations with mental disorders to reduce unnatural causes of death, such as suicide, and we also need to address the problem of natural causes of death to reduce the burden of mortality in people with mental disorders.

## Data Availability Statement

The raw data supporting the conclusions of this article will be made available as soon as all analyzes in the study are performed.

## Ethics Statement

The studies involving human participants were reviewed and approved by the Regional Committee for Medical and Health Research Ethics, Central Norway (ref. 2011/137) and conducted according to the Declaration 130 of Helsinki. The study is registered in the ClinicalTrials.gov (ref. NCT01415323). The patients/participants provided their written informed consent to participate in this study.

## Author Contributions

AP and AV designed the protocol of the study. AP and OD conducted the analysis. AV obtained funding. AV, TT, OD, KH, and AP interpreted the results. AP, OD, and AV drafted the manuscript. All authors contributed to the article and approved the submitted version.

## Funding

The study was funded by St. Olavs University Hospital and the Norwegian University of Science and Technology.

## Conflict of Interest

The authors declare that the research was conducted in the absence of any commercial or financial relationships that could be construed as a potential conflict of interest.

## Footnotes

1
http://www.ssb.no

2
https://www.fhi.no

3
https://clinicaltrials.gov/ct2/show/NCT01415323

4
https://www.fhi.no/en/hn/health-registries/cause-of-death-registry/

5
http://www.ssb.no
